# TGF-β1-Mediated Leukocyte Cell-Derived Chemotaxin 2 Is Associated With Liver Fibrosis in Biliary Atresia

**DOI:** 10.3389/fped.2022.901888

**Published:** 2022-07-14

**Authors:** Jinfeng Zhao, Xiaodan Xu, Qingyun Gou, Qipeng Zheng, Liang Ge, Lingzhi Chen, Cong Zhang, Hui Ma, Shuxiang Lin, Xiaoli Hu, Jianghua Zhan

**Affiliations:** ^1^Graduate College, Tianjin Medical University, Tianjin, China; ^2^Department of General Surgery, Tianjin Children’s Hospital, Tianjin, China; ^3^Department of Laboratory Medicine, Tianjin Children’s Hospital, Tianjin, China; ^4^Department of Pediatric Research Institute, Tianjin Children’s Hospital, Tianjin, China; ^5^Department of Pathology, Tianjin Children’s Hospital, Tianjin, China

**Keywords:** biliary atresia, leukocyte cell-derived chemotaxin 2, liver fibrosis, inflammatory response, diagnostics

## Abstract

**Objective:**

Biliary atresia (BA) presents as a severe infantile cholangiopathy disease, characterized by progressive liver fibrosis and the resulting poor prognosis. Leukocyte cell-derived chemotaxin 2 (LECT2) was proposed as the key gene associated with hepatic fibrosis in BA, but the molecular mechanism is unclear. This study aims to investigate the function of LECT2 in BA.

**Methods:**

A total of 53 patients were enrolled in this study; 36 patients with BA, and 17 control patients with cholestasis, including congenital biliary dilations, biliary hypoplasia, and inspissated bile syndrome. The role of LECT2 in BA was analyzed using histological and cytological tests. The correlation between LECT2 and infiltrating immune cells was further analyzed by bioinformatics. The analyses were conducted using correlational analyses and ROC curves.

**Results:**

LECT2 was highly expressed in infants with BA and positively related with fibrosis (0.1644 ± 0.0608 vs. 0.0779 ± 0.0053, *p* < 0.0001; *r*_*s*_ = 0.85, *p* < 0.0001). Serum levels of LECT2 showed high distinguishing features for patients with BA having an AUC of 0.95 (95% CI: 0.90–1.00). CD163 was highly expressed in the aggravation of fibrosis (0.158 ± 0.062 vs. 0.29 ± 0.078, *p* < 0.0001), and the expression of LECT2 was positively correlated with the accumulation of CD163^+^ macrophages (*r* = 0.48, *p* = 0.003). The bioinformatic analysis also showed that LECT2 was positively correlated with macrophage M2 (*r* = 0.34, *p* = 0.03). TGF-β1 and CD163 colocalized to the portal area in the livers of patients with BA. Moreover, TGF-β1 upregulated the expression of LECT2.

**Conclusion:**

LECT2 is highly expressed in both BA liver tissue and serum, and serum LECT2 is a potential diagnostic biomarker of BA. Meanwhile, TGF-β1 is secreted by macrophages to regulate LECT2 associated with BA liver fibrosis.

## Introduction

Biliary atresia (BA) is a severe infantile hepatobiliary disease characterized by biliary obliteration and progressive hepatic fibrosis, which leads to end-stage cirrhosis. The pathogenesis remains largely unknown despite some factors being well recognized, such as persistent inflammation and immune response (1). Leukocyte cell-derived chemotaxin 2 (LECT2) is a multifunctional cytokine, which is initially purified from plant hemagglutinin-activated human T-cell leukemia SKW-3 cell culture (2). It is mainly secreted by hepatocytes and is the direct target gene of β-catenin (3). LECT2 plays a crucial role in liver fibrogenesis (4). It promotes liver fibrosis in BA development by regulating TGF-β-mediated α-SMA and COLIA1 expression in LX-2 cells (5).

In addition, LECT2 is a modulator of immune and inflammatory reactions, which is related to the liver inflammatory response. LECT2 exerts functions such as hematopoietic stem cell mobilization in a macrophage-dependent manner and pro-inflammatory effects in non-alcoholic steatohepatitis (NASH) *via* macrophages (6, 7). These results suggest that there is a close relationship between LECT2 and macrophages. Macrophages can produce and activate the pro-fibrotic cytokine TGF-β1, which has a pro-fibrotic function in models of idiopathic pulmonary fibrosis (8).

Based on the above background, we propose the hypothesis that macrophages regulated LECT2 by secreting TGF-β1, thereby influencing the liver fibrosis in BA. This study aimed to explore the expression of LECT2 and its relationship with the inflammatory response in the procession of BA liver fibrosis.

## Materials and Methods

### Subjects

A total of 53 patients were collected, who were admitted to Tianjin Children’s Hospital from January 2016 to August 2020. They had complete medical records, serum specimens, frozen liver tissues, and paraffin-embedded liver tissues. Finally, 36 BA samples were defined as the BA group, and 17 infants with jaundice with other causes as the control group, including congenital biliary dilations (CBD), biliary hypoplasia (BH), and inspissated bile syndrome (IBS). All patients were finally confirmed by operation and pathological diagnosis. Serum was harvested on admission, and liver biopsy tissues were harvested during the operation.

### Immunohistochemistry Staining

Following deparaffinization, all sections were subjected to antigen retrieval for 15 min in sodium citrate (pH 6.0) at a high temperature. When all liver sections came back to room temperature, blocking was done with 5% goat serum albumin for 30 min. Then sections were incubated with primary antibodies of LECT2 (1:300, Abcam, Cambridge, MA), CD163 (working liquid, Bioss Beijing, China), respectively, at 4^°^C overnight. Then the liver sections were incubated with the horseradish peroxidase-conjugated IgG (secondary antibody) and added DAB. Finally, the nucleus was dyed with hematoxylin and mounted. The staining intensity was analyzed using Image-Pro Plus version 6.0 by scanning 5 random non-overlapping fields and capturing at × 200 in each liver section by a microscope with identical settings.

### Masson Staining and Liver Fibrosis Grading

All the liver samples harvested during the operation were fixed in 10% formalin immediately. After dehydration, all samples were paraffin-embedded and sliced into 4-μm slices. Masson staining for histological assessment and two specialized pathologists evaluated all liver fibrosis scores by METAVIR scoring (9). Liver fibrosis was divided into four stages according to this scoring system: F0, no fibrosis; F1, portal fibrosis without linkage; F2, fibrous septa portal-to-portal; F3, fibrous septa portal-to-portal and portal-to-central; and F4, cirrhosis. F1 and F2 stages were considered mild fibrosis group, and F3 and F4 severe fibrosis group.

### Real-Time Quantitative Polymerase Chain Reaction (RT-qPCR)

Frozen liver tissues were used to extract total RNA with the kit according to the instructions provided by the manufacturer (Tian Gen, Beijing, China) and tested the purity with NanoDrop. The extracted RNA had an absorbance 260/280 ratio of 1.9–2.0. First-strand cDNA was synthesized using the RT reagent kit including gDNA Eraser (Sangon Biotech, Shanghai, China). The expression of LECT2 was measured using the SYBR Green Master Mix kit (SYBR Premix Ex Ta q™ II, Sangon Biotech) on a Light Cycler 480 Real-Time PCR detection system. The internal reference is the glyceraldehyde 3-phosphate dehydrogenase (GAPDH). The primer sequences are listed in Table 1.

**TABLE 1:** Primer sequences used in this study.

Name	Primer
LECT2	F: 5′-GTGCTGGCAAGTCTTCCAATGAG-3′
	R: 5′-CCAGTGAATGGTGCGTACACAG-3′
GAPDH	F: 5′-GGGTGATGCTGGTGCTGAGTATGT-3′
	R: 5′-AAGAATGGGAGTTGCTGTTGAAGT-3′

*LECT2, leukocyte cell-derived chemotaxin 2; GAPDH, glyceraldehyde 3-phosphate dehydrogenase; F, forward primer; R, reverse primer.*

### Enzyme-Linked Immunosorbent Assay

Leukocyte cell-derived chemotaxin 2 level of the serum collected at the time of admission was detected using enzyme-linked immunosorbent assay (ELISA) kit (RenjieBio, Shanghai, China) according to the protocols provided by the manufacturer. Briefly, the peroxide-conjugated anti-LECT2 (100 μl/well) antibody was added to the microplate with a standard or serum sample (50 μl/well) and incubated at 37.0^°^C for 1 h. After washing three times, the peroxidase-specific substrate was added to each well and incubated at 37.0^°^C for 15 min. After that, a termination solution (50 μl/well) was added to stop the reaction. Finally, the color intensity proportional to the concentration of LECT2 was achieved at 450 nm.

### Bioinformatics Analysis

The biliary atresia expression profile datasets GSE15235 were downloaded from the Gene Expression Omnibus^1^ database. GSE15235 included a total of 47 biliary atresia liver tissues, and the raw data were preprocessed with the “affy” package (10) in R software (version 4.1.2).^2^ The gene expression matrix data were processed using the CIBERSORT method (11) in the “IOBR” package (12) to obtain the infiltration matrix for 22 immune cell types. Finally, Spearman correlation analysis of LECT2 and infiltrating immune cells was performed.

### Immunofluorescence

Immunofluorescent staining was similar to the immunohistochemistry staining (IHC) protocol, the sections of liver tissues were incubated with primary rat monoclonal anti-CD163 and rabbit polyclonal anti-TGF-β1 (1:300, Bioss Beijing, China) at 4^°^C overnight, and washed completely in phosphate buffer solution (PBS), with the secondary antibodies combined with Alexa488/594 (Bioss Beijing, China) for 2 h. Sections were mounted for observation under the microscope after being washed.

### Cell Culture and Treatment

Human normal liver cell line HL-7702 (L-02) (BNCC351907) was purchased from bnbio (BNCC351907, China) and cultured in 1640 DMEM medium (SH30809.01, Jiangsu KeyGEN BioTECH, China), containing 10% fetal bovine serum medium. All cells were incubated in a humidified atmosphere of 5% CO_2_ at 37°C. L-02 cells were treated with PBS and 100 ng/ml recombinant human TGF-β1 (rh TGF-β1, Bioss Beijing, China), and rhTGF-β1 plus ICG001 (inhibits TCF/β-catenin/CBP mediated transcriptional activity) for 24 h and measured the expression of LECT2.

### Data Analysis

The data were analyzed using SPSS 22.0, and non-normal distribution data were expressed as the median. The chi-square test was used to evaluate qualitative data (gender). The unpaired *t* or Mann–Whitney test was used to evaluate quantitative data in two groups. Comparison between multiple groups was performed by one-way analysis of variance. The correlations were analyzed by Pearson coefficient analysis. The area under the receiver operating characteristic (ROC) curve (AUC) was calculated, and the optimal cutoff values of serum LECT2 were determined. The *p*-value < 0.05 was considered to be statistically significant.

## Results

### Baseline Characteristics

A total of 53 patients were enrolled in this study. Among them, 36 patients, including 20 boys and 16 girls, had BA and the median age was 53.0 days, and 17 patients, including 8 boys and 9 girls, had no BA, and the median age was 89.0 days. Gender was tested using the chi-square test and χ^2^ = 0.335, *p* = 0.56. There was no statistical difference in gender, but the age was statistically significant (*p* = 0.02). Beyond CBD, there was no statistical difference in BA, BH, and IBS (*p* = 0.61; Table 2).

**TABLE 2:** Demographic characteristics of study subjects.

Projects		BA			Control		*P*
*Gender (M/F)		20/16			8/9		0.56
^#^Age (days)		53.0 (39.0, 68.0)			89.0 (49.0, 921.0)		0.02

	**BA (36)**		**Control (17)**				***P***
			**CBD**	**BH**		**IBS**	

*Gender (M/F)	20/16		1/5 (29.4%)	4/1 (35.3%)		3/3 (35.3%)	0.56
^#^Age (days)	53.5 (40.5, 67.8)		1284.5 (763.5, 1919.5)	106.0 (54.5, 123.0)		43.5 (29.0, 70.0)	0.02

Gender was tested using the chi-square test and χ^2^ = 0.335; all other rows were tested using the Mann–Whitney U-test. *M/F (percent); ^#^Median (interquartile range). BA, biliary atresia group; control, non-BA group; CBD, congenital biliary dilation; BH, biliary hypoplasia; IBS, inspissated bile syndrome; M/F: male/female.

### Leukocyte Cell-Derived Chemotaxin 2 Was Highly Expressed in Biliary Atresia Liver Tissues and Associated With Liver Fibrosis

**FIGURE 1:**
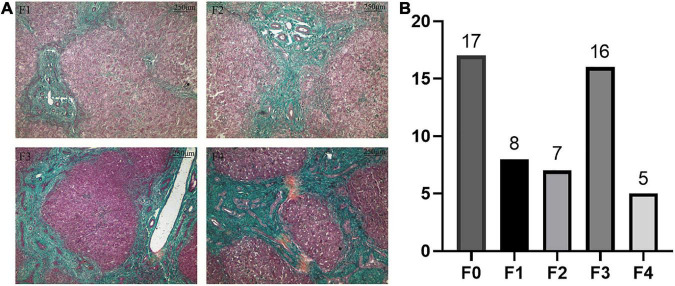
**(A)** Masson staining in liver tissues of patients with BA. **(B)** The distribution of liver fibrosis. **(A)** Liver fibrosis levels were accessed by Masson staining. Collagen fibers are blue, and cytoplasm is red. F1: portal fibrosis without linkage; F2: fibrous septa portal-to-portal; F3: fibrous septa portal-to-portal and portal-to-central; and F4: cirrhosis. **(B)** The distribution of liver fibrosis levels in 53 patients. Including 17 cases with F0 (no fibrosis), eight cases with F1, seven cases with F2, 16 cases with F3, and five cases with F4.

**FIGURE 2:**
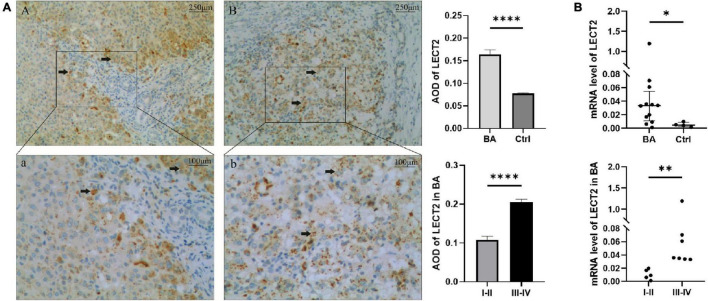
**(A)** The expression of LECT2 by IHC. **(B)** The expression of LECT2 by qPCR. **(A)** The expression of LECT2 in liver tissues of patients with mild liver fibrosis **(Aa)** and patients with severe liver fibrosis **(Bb)** in the BA group by immunohistochemical staining. A and a: The black arrows point to the area of positive expression, which concentrate on the edge of portal area. **(Bb)** The positive expression spreads the whole hepatic lobule (**A,B,** 40×; a,b, 100 ×). A stronger AOD was observed in the BA group than the control group. In patients with BA, a stronger AOD was observed in the severe liver fibrosis than the mild liver fibrosis. **(B)** Determining the LECT2 expression at the transcriptional level. The LECT2 mRNA expression in the BA group was higher than the control group, and in patients with BA, the severe liver fibrosis group was higher than the mild liver fibrosis group. LECT2, leukocyte cell-derived chemotaxin 2; AOD, average optical density; I–II, F1/F2; III–IV, F3/F4. *p < 0.05, ^**^p < 0.01, ^****^p < 0.0001.

According to Masson staining, no fibrosis occurred in the control group, 15 patients with mild fibrosis and 21 patients with severe fibrosis in the BA group (Figures 1A,B). The immunohistochemical result showed that positive staining of the maker was localized in granular form in the cytoplasm of hepatocytes mainly and distributed scattered in the portal area, and gradually spread from the portal area to the whole hepatic lobule with the aggravation of liver fibrosis. In contrast, the protein was expressed less in the liver tissues of the control group. A stronger average optical density (AOD) of IHC staining was observed in the BA group for LECT2 than in the control group (0.1644 ± 0.0608 vs. 0.0779 ± 0.0053, *p* < 0.0001; Figure 2A). Together, LECT2 was highly expressed in the patients with BA and positively correlated with fibrosis degree (*r*_*s*_ = 0.85, *p* < 0.0001). The extracted total RNA was used to determine LECT2 expression at the transcriptional level. Consistent with histological findings, livers from BA subjects had higher LECT2 mRNA expression compared to the control group [0.0334 (0.110, 0.547) vs. 0.0047 (0.0024, 0.0087), *p* < 0.05; Figure 2B].

### Leukocyte Cell-Derived Chemotaxin 2 Was a Potential Diagnostic Biomarker for Biliary Atresia

**FIGURE 3:**
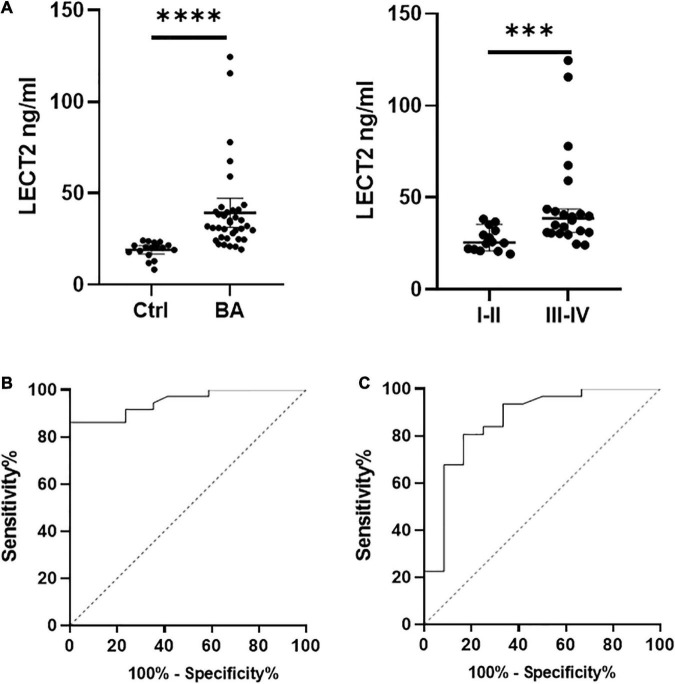
**(A)** The serum levels of LECT2. **(B)** The ROC of LECT2. **(C)** The ROC of GGT. **(A)** The serum levels of LECT2 in 53 patients. Ctrl: The non-BA infants with jaundice with other causes, including congenital biliary dilations, biliary hypoplasia, and inspissated bile syndrome. The serum levels of LECT2 in the BA group were higher than the control group. In BA group, the LECT2 serum level in patients with severe liver fibrosis was higher than that in those with mild liver fibrosis. LECT2, leukocyte cell-derived chemotaxin 2, I–II, F1/F2; III–IV, F3/F4. **(B)** The ROC of LECT2 in diagnosing BA. The AUC was 0.95 (95% CI: 0.90–1.00), and the cutoff value was 23.99 ng/ml with a sensitivity of 86% and specificity of 94%. **(C)** The ROC of GGT in diagnosing BA. The AUC was 0.86 (95% CI: 0.73–1.00), and the cutoff value was 797.5 U/L with a sensitivity of 23% and specificity of 92%. ^***^p < 0.001, ^****^p < 0.0001.

Timely and effective diagnostic approaches are needed for diagnosing BA. However, existing indicators are not universally applicable because of the high cost and lack of uniform indicators (13, 14). The serum LECT2 level was significantly higher in patients with BA compared with the control group [32.03 ng/ml (25.42, 40.47) vs. 20.37 ng/ml (17.07, 22.37), *p* < 0.0001; Figure 3A]. Also, in patients with BA, the severe fibrosis group was higher than the mild fibrosis group [38.63 ng/ml (30.97, 47.57) vs. 25.58 ng/ml (21.66, 32.89), *p* < 0.001; Figure 3A]. In particular, the serum levels of five F4 patients were extremely high. Therefore, LECT2 was able to distinguish BA from other cholestasis with an AUC of 0.95 (95% CI: 0.90–1.00), and the cutoff value was 23.99 ng/ml with a sensitivity of 86% and specificity of 94% (Figure 3B). It has absolute advantages over GGT, commonly used liver function index, and the AUC of GGT was 0.86 (95% CI: 0.73–1.00; Figure 3C).

### Leukocyte Cell-Derived Chemotaxin 2 Participated in the Inflammatory Response and Was Upregulated by TGF-β1

**FIGURE 4:**
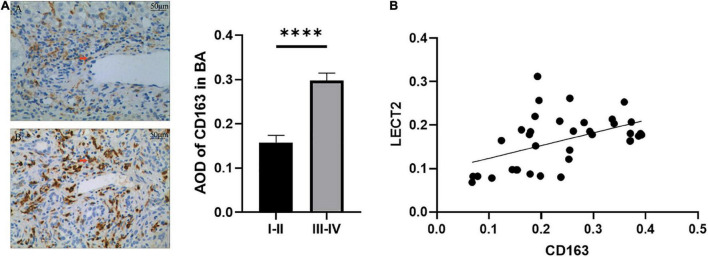
**(A)** The expression of CD163 by IHC. **(B)** The relationship between CD163 and LECT2. **(A)** The expression of CD163 in patients with mild liver fibrosis **(A)** and patients with severe liver fibrosis **(B)** of the BA group. The red arrows point to the area of positive expression (200×). A stronger AOD of CD163 was observed in patients with severe liver fibrosis than those with mild liver fibrosis through semi-quantitative analysis. **(B)** LECT2 was positively correlated with CD163. LECT2, leukocyte cell-derived chemotaxin 2; AOD, average optical density; I–II, F1/F2; III–IV, F3/F4. ^****^p < 0.0001.

**FIGURE 5:**
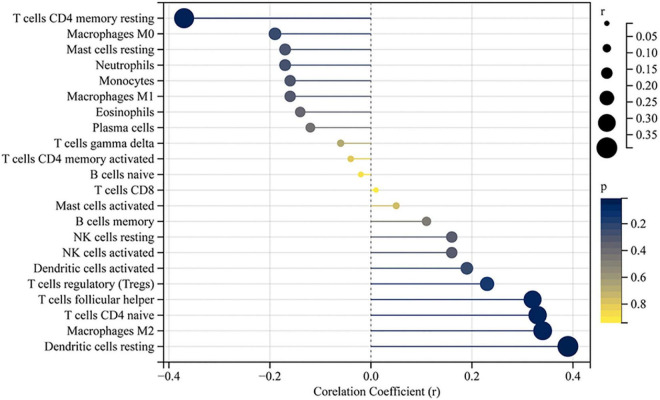
Correlation between LECT2 and infiltrating immune cells. The size of the dots represents the strength of the correlation between LECT2 and immune cells; the larger the dots, the stronger the correlation, and the smaller the dots, the weaker the correlation. The color of the dots represents the *p*-value; the bluer the color, the lower the *p*-value, and the yellower the color, the larger the *p*-value. The *p*-value < 0.05 was considered statistically significant.

**FIGURE 6:**
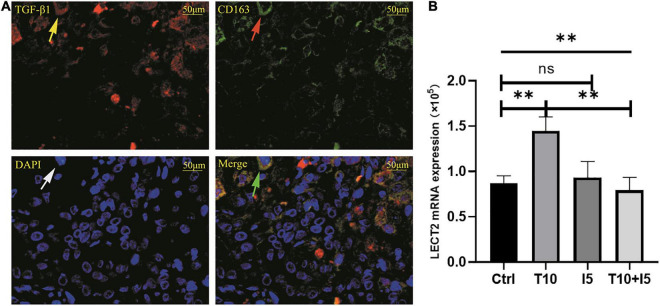
**(A)** TGF-β1 and CD163 were co-localized in BA livers. **(B)** rhTGF β1 upregulated the expression of LECT2. **(A)** The red fluorescence indicated by the yellow arrow is TGF-β1, CD163 was labeled by green fluorescent indicated by the red arrow, all nucleus were blue fluorescent indicated by the white arrow. The green arrow points to the merge of TGF-β1 and CD163. **(B)** The human normal liver cells (L02) were conducted *in vitro* and divided into four groups, namely, Ctrl, T10, I5, and T10 + I5. The differences among the four groups were statistically significant by one-way ANOVA. In other words, rhTGF β1 upregulated the expression of LECT2. However, the effect was reversed with the use of LECT2 blocker ICG001. LECT2, leukocyte cell-derived chemotaxin 2; Ctrl, blank control group; T5, rh TGF-β1 5 ng/ml; T10, rh TGF-β1 10 ng/ml; I5, ICG 001 5 mg/ml; T10 + I5, rh TGF-β1 10 ng/ml + ICG 001 5 mg/ml. ^**^p < 0.01.

The accumulation of inflammatory cells increased with the development of fibrosis in patients with BA. Meanwhile, CD163^+^ macrophages were mainly accumulated in the portal area, and it was highly expressed in the aggravation of fibrosis (0.158 ± 0.062 vs. 0.29 ± 0.078, *p* < 0.0001; Figure 4A). Interestingly, this is similar to the features of LECT2, which are distributed scattered in the portal area. And the expression of LECT2 was positively correlated with the accumulation of CD163^+^ macrophages (*r* = 0.48, *p* = 0.003; Figure 4B). Bioinformatic correlation analysis also showed that LECT2 was positively correlated with macrophages M2 (*r* = 0.34, *p* = 0.03; Figure 5). TGF-β1 and CD163 colocalized in the portal area in the livers of patients with BA with immunofluorescence (IF) (Figure 6A). Besides, the human normal liver cells were conducted *in vitro*. LECT2 mRNA level of the L-02 cells treated with recombinant human TGF-β1 (rh TGF-β1) was higher than those treated with PBS. After treatment with ICG001, which targets the cAMP-response element-binding protein, reversed this effect (Figure 6B). Therefore, the TGF-β1 derived from CD163^+^ macrophages in the portal area of BA livers could upregulate the expression of LECT2 through the cAMP-response element-binding protein.

## Discussion

BA presents as a severe hepatobiliary disease in newborns with a difficult diagnosis and poor prognosis because of its unclear pathogenesis. If untreated, biliary cirrhosis leads to death by age 2 years (15). Therefore, clarifying the mechanism of progressive liver fibrosis is one of the urgent problems to be solved. However, a breakthrough in liver fibrogenesis was published in the journal *Cell*. LECT2 as a crucial ligand for endothelial cell-specific orphan receptor Tie1 promotes sinusoid capillarization and inhibits portal angiogenesis to exacerbate fibrosis (4). As is known to all, angiogenesis plays an important role in liver fibrosis, and the hepatic subcapsular spider-like telangiectasis sign is a specific marker for BA (16). We believe that it may be related to the mechanism of BA fibrosis. Moreover, it was proposed to be related to liver fibrosis in BA and involved in BA development (5).

Thus, we initially evaluated the expression of LECT2 in liver tissue and serum of patients with BA. In this study, LECT2 was initially distributed scattered in the portal area and gradually spread to the whole hepatic lobule with the aggravation of liver fibrosis, which possibly resulted from its function as a leukocyte cell-derived chemotaxin. Both IHC and qPCR results confirmed that the expression of LECT2 was significantly elevated in BA liver tissues, and its expression was positively correlated with the degree of liver fibrosis. Further ELISA testing revealed that serum LECT2 levels were also consistent with these results, suggesting that LECT2 is an important factor associated with liver fibrosis in BA.

In this study, we also suggested that LECT2 is a novel biomarker for the identification of BA, which is achieved by the sensitivity to fibrosis. And the cutoff value is 23.99 ng/ml. Some biomarkers have already been proposed, but LECT2 has obvious advantages over GGT according to the AUC in our findings, and the GGT as the diagnostic test must be considered the age (17). In addition, MMP7, one of the biomarkers of BA, has high credibility (18). Jiang et al. (14) reported the cutoff value of MMP7 as 10.37 ng/mL, while Yang et al. (13) reported the cutoff value as 52.85 ng/ml. Cost is also a critical aspect of MMP7. More universally applicable diagnostic methods are needed, and serum LECT2 has a potential value in BA diagnostics.

After demonstrating the relationship between LECT2 and BA liver fibrosis, we further investigated whether LECT2 is involved in the inflammatory response to influence the liver fibrosis process. Some scholars propose that the central part of BA pathogenesis is immune dysregulation (19). The presence of chemotaxis inflammatory cells provides additional information on LECT2 in BA from immunological perspectives. LECT2 has specific chemotactic effects on neutrophils, monocytes, and macrophages (20). We focused on its chemotactic effects associated with macrophages, a vital kind of inflammatory cell with remarkable heterogeneity, which play a crucial role in liver homeostasis and disease (21). Various stress lead to sustained hepatic inflammation, which results in the recruitment of blood monocytes that infiltrate the liver and promote the development of fibrosis (22). Moreover, macrophages enhance the survival of myofibroblasts, which is essential for the development of liver fibrosis (23). And the polarization of macrophages infiltrating the liver is associated with liver fibrosis in patients with BA (24). In particular, CD163^+^ macrophages act as anti-inflammatory and promote fibrosis. The results revealed that LECT2 and CD163 + macrophages have a mutually promoting role in BA liver fibrosis.

So, in what way does LECT2 interact with CD163^+^ macrophages in the process of BA liver fibrosis?

On the one hand, LECT2 stimulates the activation and recruitment of CD163^+^ macrophages in the BA liver. Local inflammatory reactions persist in the liver caused by BA, which leads to blocking the degradation of the extracellular matrix. On the other hand, macrophages produce pro-fibrotic mediators such as TGF-β1 and platelet-derived growth factor to regulate the hepatic stellate cells trans-differentiation to the key extracellular matrix producing cells, myofibroblasts (25). And LECT2 participated in the inflammatory response to promote liver fibrosis and was regulated by TGF-β1 (5). Therefore, we hypothesize that TGF-β1 is the link between LECT2 and CD163 + macrophage interactions. In addition, it was noted that CD163^+^ macrophages secreted TGF-β1 in the BA livers by immunofluorescence staining. TGF-β1 upregulated LECT2 in the cell experiments. Altogether, the immunological significance of LECT2 is correlated with macrophage infiltration in the portal area in BA. LECT2 interacts with CD163^+^ macrophages through TGF-β1 in the fibrogenesis of BA livers.

### Limitation

This study is a single-center study, and the number of cases needs to be expanded and validated prospectively.

## Conclusion

LECT2 is highly expressed in both BA liver tissue and serum, and serum LECT2 is a potential diagnostic biomarker of BA. Meanwhile, TGF-β1 is secreted by macrophages to regulate LECT2 associated with BA liver fibrosis.

## Data Availability Statement

The original contributions presented in this study are included in the article/supplementary material, further inquiries can be directed to the corresponding author.

## Ethics Statement

The studies involving human participants were reviewed and approved by the Ethics Committee of Tianjin Children’s Hospital and the certificate number is 2021-YKY-01. Written informed consent to participate in this study was provided by the participants’ legal guardian/next of kin.

## Author Contributions

JFZ, XX, QG, QZ, LG, LC, and CZ: contributed to the design and implementation of the research, aided in choosing the patients, read, and approved the final manuscript. HM, SL, XH, and JHZ: supervised the findings of this work, discussed the results, read, and approved the final manuscript. All authors contributed to the article and approved the submitted version.

## Conflict of Interest

The authors declare that the research was conducted in the absence of any commercial or financial relationships that could be construed as a potential conflict of interest.

## Publisher’s Note

All claims expressed in this article are solely those of the authors and do not necessarily represent those of their affiliated organizations, or those of the publisher, the editors and the reviewers. Any product that may be evaluated in this article, or claim that may be made by its manufacturer, is not guaranteed or endorsed by the publisher.

## Footnotes

1
https://www.ncbi.nlm.nih.gov/geo/

2
http://r-project.org/
